# MIRAGE: a functional genomics-based approach for metabolic network model reconstruction and its application to cyanobacteria networks

**DOI:** 10.1186/gb-2012-13-11-r111

**Published:** 2012-11-29

**Authors:** Edward Vitkin, Tomer Shlomi

**Affiliations:** 1 Department of Computer Science, Technion - Israel Institute of Technology, Haifa 32000, Israel

## Abstract

Genome-scale metabolic network reconstructions are considered a key step in quantifying the genotype-phenotype relationship. We present a novel gap-filling approach, MetabolIc Reconstruction via functionAl GEnomics (MIRAGE), which identifies missing network reactions by integrating metabolic flux analysis and functional genomics data. MIRAGE's performance is demonstrated on the reconstruction of metabolic network models of *E. coli *and *Synechocystis *sp. and validated via existing networks for these species. Then, it is applied to reconstruct genome-scale metabolic network models for 36 sequenced cyanobacteria amenable for constraint-based modeling analysis and specifically for metabolic engineering. The reconstructed network models are supplied via standard SBML files.

## Background

Genome-scale metabolic network reconstructions are considered a key step in quantifying the genotype-phenotype relationship [1]. While the process of manually reconstructing genome-scale metabolic network models is complex [2], such networks have already been manually reconstructed for more than 50 organisms [3], including common model microorganisms [4,5], industrially relevant microbes [6-9], various pathogens [10-13], and recently for human cellular metabolism [14]. A modeling approach called constraint-based modeling serves to analyze the function of such networks by solely relying on simple physical-chemical constraints [15,16] and is frequently used to predict various phenotypes of microorganisms (reviewed in [3,17-20]). Several applications of constraint-based modeling for metabolic engineering of microbial species has been presented (reviewed in [17,21]).

The two major computational challenges in metabolic network reconstruction are (i) the identification of missing reactions in a metabolic network, and (ii) the association of genes with network reactions. The identification of missing reactions, referred to as 'gap-filling', is commonly performed based on a pre-defined metabolic capability that the network is expected to be able to fulfill. For example, the capability to generate essential biomass products under various genetic and environmental conditions [22-25], the synthesis of specific compounds identified via metabolomics [26], the flow of specifically measured flux rates [27], and the ability to activate a core set of reactions under a steady-state assumption [28]. Missing reactions are obtained either from other species [22,23,26-28], or via computational chemistry methodologies that enumerate possible metabolic routes [29], aiming to identify a minimal number of missing reactions to fulfill the required objective. A specific approach for network reconstruction that is based on the concept of elementary flux modes [30] was previously applied to successfully recover missing network reactions [16,31,32]. Another gap-filling approach that integrates some of these principles has been recently used to reconstruct 130 genome-scale metabolic network models of various bacteria [22]. While most of the above gap-filling methods rely strictly on metabolic flux analysis and do not utilize functional genomics data to guide the search for missing reactions, computational methods that aim to address the second challenge of gene-reaction assignment do rely intensively on functional genomics data. Specifically, several methods predict gene assignment based on genomic data, utilizing principles such as conserved chromosomal proximity [31,33,34] and similarity in phylogenetic profiles with neighboring genes in the same pathway [32,35,36]. Others rely on an additional array of functional genomics data, including gene co-expression and protein-protein interactions [37-43].

Here, we present a novel approach, MetabolIc Reconstruction via functionAl GEnomics (MIRAGE), for reconstructing metabolic network models and specifically addressing the problem of gap-filling, by searching for missing reactions whose presence is supported by various functional genomic data. Specifically, to reconstruct a metabolic network model for an organism of interest, MIRAGE starts from a core set of reactions, whose presence is established via strong genomic evidence, and identifies missing reactions that are required to activate the latter core reactions (in addition to biomass requirement) by identifying additional reactions, whose presence is further supported by phylogenetic profiles and gene expression data. The performance of MIRAGE, in comparison to previous methods, is demonstrated on the reconstruction of network models for *Escherichia coli *and the cyanobacteria *Synechocystis *sp. PCC 6803, validated via existing networks for these species. Then, it is applied to reconstruct genome-scale metabolic network models for 36 sequenced cyanobacteria (supplied via standard Systems Biology Markup Language (SBML) files [44]), amenable for constraint-based modeling analysis and specifically for metabolic engineering. To demonstrate the utility of the reconstructed cyanobacteria networks, a strain design method was applied to predict gene knockouts whose implementation is expected to significantly elevate the production rate of an important nutritional product, astaxanthin.

## Results and discussion

### MIRAGE

MIRAGE is a functional genomics-based model reconstruction approach that aims to generate a genome-scale metabolic network model for an organism of interest, given a core set of reactions that are known to exist in its network, and optionally, a definition of a biomass reaction. The core set of reactions can be automatically derived strictly from genomic data, based on strong sequence similarity with known enzyme-coding genes in other species. The method then aims to find missing reactions (from a universal database of candidate gap-filling reactions such as the Kyoto Encyclopedia of Genes and Genomes (KEGG)), supported by functional genomics data, whose addition to the network would lead to a functional model. The method follows a two-step procedure, starting with the utilization of functional genomics data to estimate the probability of including each reaction from the universal database in the reconstructed network, and then, metabolic flux analysis that selects the most likely set of reactions whose addition to the network would satisfy the above described objectives.

For the first step, we utilize two functional-genomics data sources to estimate the likelihood that a reaction from a universal reactions database should be included in the target metabolic network: (i) enzymes' phylogenetic profiles, and (ii) gene expression. Specifically, we define a weight for each reaction in the universal database (that is not already included in the reconstruction's core reactions set), based on the functional similarity between neighboring enzymes, in terms of resemblance of phylogenetic profiles, and correlation in gene expression of the enzyme-coding genes (Materials and methods).

Enzyme phylogenetic profiles were extracted from KEGG, representing a pattern of enzyme presence or absence across an available collection of species. For each reaction in KEGG, we computed a phylogenetic weight, representing the likelihood for its inclusion in the network reconstruction. Specifically, the phylogenetic weight of a certain reaction is calculated based on the maximal Jaccard coefficient between its phylogenetic profile and the corresponding profiles of its neighboring core reactions in the network (Materials and methods). Similarly, an expression weight for each reaction was calculated by evaluating gene expression profiles (measured in the target organism) of potential enzyme-coding genes (considering all non-annotated genes in the genome), compared with the expression profiles of known genes associated with neighboring core reactions. The sum of the phylogenetic and expression weights after proper normalization was used as input for the second reconstruction step (Materials and methods).

The second reconstruction step aims to find a set of high weight gap-filling reactions that satisfy the objectives described above. Towards this goal, we employed the following reaction pruning procedure. Starting from a metabolic network model consisting of all reactions in the universal reaction database, we iteratively remove potential gap-filling reactions, as long as the removal does not affect the consistency of the model. In each iteration, the probability of choosing a certain reaction for removal is inversely proportional to its weight - that is, low weight reactions have a higher probability to be chosen first for removal. The model consistency check procedure involves the usage of constraint-based modeling to verify that the remaining network (i) enables each core reaction to carry non-zero metabolic flux within a stoichiometrically balanced flux distribution, accounting for reaction directionality constraints, (ii) enables the production of all essential biomass constituents, and (iii) accounts for the growth-associated dilution of all network metabolites (that is, guaranteeing that the network consists of complete pathways for either the transport or *de novo *synthesis of all metabolites that exist in the network) [45]. Since the reactions' scanning order may affect the resulting model, the algorithm is executed several times with different, random pruning orders (Materials and methods). The fraction of obtained models that contains a certain reaction reflects the confidence that it should be included in the final model. Hence, to construct the final metabolic network model, we run the reactions removal procedure again, based on an ordering defined by the received confidence values (Materials and methods).

Notably, the presented method extends upon the Model Building Algorithm (MBA) of Jerby *et al. *[28] that was recently used to reconstruct a model of human liver metabolism. The MBA method addresses only the first objective from the above list, while not accounting for biomass production and growth-associated metabolite dilution, which are of less importance for the modeling of human tissue metabolism. Furthermore, it accounts for functional genomics data in a more limited manner, by using them only to define two core sets of reactions with either a moderate or high probability to be retained in a specific tissue model. In contrast, MIRAGE assigns a continuous score per each reaction that reflects its probability to be retained in a specific species model, allowing us to make better use of these data.

The described method is computationally demanding since each trial of the random reaction pruning procedure (out of the 500 trials performed to gather sufficient confidence statistics), requires eliminating each reaction from the universal reactions set in turn, and checking the consistency of the resulting model. Implementing the speedup heuristic suggested by Jerby *et al. *[28], which aims to minimize the number of linear optimizations required in each model consistency check, provided some improvement in running time. However, each random pruning trial still took around 35 hours, which made the entire method computationally intractable. The significant increase in running time in comparison to the method of Jerby *et al. *resulted from the markedly large size and complexity of the universal reaction database in comparison to the human network model used by Jerby *et al*., and the additional reconstruction objectives previously not accounted for.

To overcome this, we implemented the following additional speed-up techniques (Materials and methods). First, the model consistency check procedure is based on identifying a set of flux distributions in which all core reactions are activated (that is, have non-zero flux), and is applied following the removal of each reaction in the reaction pruning procedure. The first speed-up involved the utilization of flux distributions computed in one call to the model consistency check procedure in subsequent calls to this procedure (testing the potential removal of subsequent reactions in the pruning order) to avoid time-consuming linear programming optimizations. Second, to further minimize the number of performed linear optimizations, the latter are now formulated with the objective of minimizing flux through subsequent gap-filling reactions in the pruning order. These two speed-up techniques, significantly elaborated upon in Additional file 1, provide a 100-fold improvement in running time.

**Figure 1:**
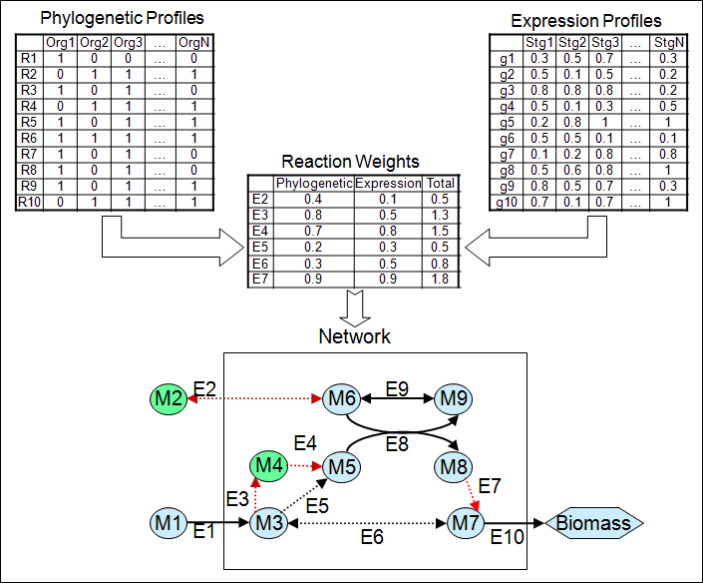
**The application of MIRAGE on a toy model**. Core reactions (E1, E8, E9 and E10) are marked with straight lines, while gap-filling reactions are marked with dashed lines. A weight for each reaction is computed based on the correlation of its phylogenetic and expression profiles with those of neighboring core reactions in the network. Reactions predicted for gap-filling by MIRAGE are in red. Specifically, E2 is chosen to enable the growth-associated dilution of metabolites M6 and M9. E3 and E4 are chosen (instead of E5, which has a significantly lower weight) to enable the flux activation of E8 and E9. E7 is chosen to enable flux activation of E8 and E9 under steady-state. Reaction E6 is not chosen for gap-filling as it is redundant given the above-mentioned chosen essential reactions.

Figure 1 illustrates the working of MIRAGE on a toy model. Reactions E1, E8, E9 and E10 are core reactions, while all the other reactions are candidates for gap-filling. MIRAGE predicts the addition of reactions E2, E3, E4 and E7 to enable flux activation of all core reactions, biomass production, and accounting for growth dilution of all metabolites in the core. The inclusion of reactions leading from M3 to M5 is required to enable flux activation of core reactions E8 and E9. In this case, the choice of including both reactions E3 and E4 for gap-filling, instead of the single reaction E5, is based on higher support for the former reactions in the functional-genomic data. Reaction E2 is predicted for gap-filling to compensate for growth-associated dilution of metabolites M6 and M9 [45].

### Validation of MIRAGE in the reconstruction of a metabolic network for *E. coli*

To evaluate the performance of MIRAGE, we applied it to reconstruct a metabolic network model for *E. coli*, for which a comprehensively curated model (iAF1260) is already available for validation [46]. Towards this end, we extracted a cross-species reactions dataset from KEGG having 7,211 reactions (referred to as the universal reactions set). To define a core set of known *E. coli *reactions to be used by MIRAGE, we considered KEGG reactions annotated as existing in *E. coli *and also belonging to iAF1260, plus the known biomass and all exchange reactions from iAF1260. Then we removed dead-end reactions that cannot be activated within a feasible flux distribution when considering the entire universal reactions set, yielding a core set of 812 reactions. Performing standard flux variability analysis [47] when focusing only on this set of 812 core reactions revealed that 45% (365/812) of these reactions are on dead-ends. MIRAGE's task is hence to identify gap-filling reactions that would resolve these dead-ends, aiming to identify a remaining set of 109 reactions from iAF1260. Notably, our analysis did not account for subcellular localization of metabolic processes, and hence duplicated reactions in iAF1260 that correspond to multiple compartments were removed.

**Figure 2:**
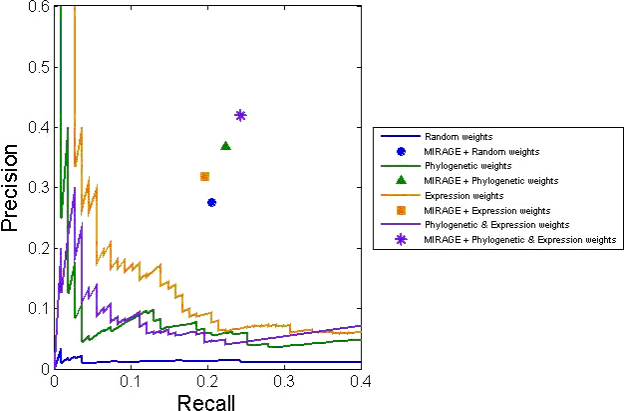
**MIRAGE's predictive performance on reconstructing a known metabolic network of *E. coli***. The precision and recall of MIRAGE is marked with a star symbol. The precision and recall of several controls, including variants of MIRAGE that utilize only phylogenetic data, only expression data, or no functional-genomics data, are marked with a triangle, bar, and circle, respectively. The predictive performance of the functional genomic data (that is, by ordering potential gap-filling reactions based on their computed functional genomic weights, without utilizing metabolic flux analysis) is shown by the straight lines: the performance of the phylogenetic data, gene expression, and both data sources are colored green, yellow, and purple, respectively. The performance of random predictions of gap-filling reactions is colored blue.

Comparison of MIRAGE's reconstructed network model for *E. coli *with iAF1260 shows a predictive precision of 41.9% and recall of 24.3%, which is significantly better than random sampling of gap-filling reactions (hyper-geometric *P*-value <10^-16^; Figure 2; Additional file 1, part 6, and Supp. Table 1 in Additional file 1). As controls, we assessed the predictive performance of using only the functional genomics data based on the computed reaction weights (by ordering potential gap-filling reactions based on their computed weights), and the predictive performance of MIRAGE without utilizing functional genomics data (by assigning reactions with random weights; as done in the MBA algorithm). Using only the functional genomics data, the resulting predictive performance was significantly lower than that of MIRAGE (Figure 2), reaching a precision of 6.1%, under a recall level of 19.6% (*P*-value = 2 × 10^-9^). Without utilizing functional genomics data, the predictive performance was also markedly lower, with a precision of 27.5% and recall of 20.6% (*P*-value <10^-16^). Using only gene expression [48] or phylogenetic weights (based on all species in KEGG) provided lower precision of 31.8% and 36.9%, respectively, with slightly lower recall levels (19.6% and 22.4%, respectively) to those achieved when utilizing both (Figure 2), demonstrating the importance of integrating multiple functional-genomics data sources. As a further control, we applied MIRAGE to reconstruct a metabolic network model for *E. coli*, without prior knowledge of exchange reactions (which in the above analysis were taken from the model of iAF1260), finding an overall similar predictive performance, showing an improvement of MIRAGE compared to other approaches (Supp. Table 2 in Additional file 1).

**Table 1:** Predicted knockout strategies for Astaxanthin over-production in cyanobacteria

Organism	Growth rate (mutant/wt) [h^-1^]	Astaxanthin production [μmol gDW^-1 ^h^-1^] (min/max)	Knockout reaction names	KEGG reaction ID/EC number
*P. marinus *as9601	0.026/0.061	2.18/2.18	Dimethylallyl-diphosphate: isopentenyl-diphosphate dimethylallyl transtransferase	R01658/EC 2.5.1.1
			5-O-(1-Carboxyvinyl)-3-phosphoshikimate phosphate-lyase	R01714/EC 4.2.3.5
*T. elongatus*	0.016/0.048	2.08/2.08	Dimethylallyl-diphosphate: isopentenyl-diphosphate dimethylallyl transtransferase	R01658/EC 2.5.1.1
			Phosphoenolpyruvate: D-erythrose-4-phosphate C-(1-carboxyvinyl)transferase	R01826/EC 2.5.1.54
*G. violaceus*	0.033/0.061	1.64/1.64	Succinate:(acceptor) oxidoreductase	R00408/EC 1.3.99.1
			Dimethylallyl-diphosphate: isopentenyl-diphosphate dimethylallyl transtransferase	R01658/EC 2.5.1.1
*Synechococcus *pcc7942	0.033/0.061	1.64/1.64	Succinate:(acceptor) oxidoreductase	R00408/EC 1.3.99.1
			Dimethylallyl-diphosphate: isopentenyl-diphosphate dimethylallyl transtransferase	R01658/EC 2.5.1.1
Cyanobacteria cyb	0.032/0.058	1.57/1.57	Succinate:(acceptor) oxidoreductase	R00408/EC 1.3.99.1
			Dimethylallyl-diphosphate: isopentenyl-diphosphate dimethylallyl transtransferase	R01658/EC 2.5.1.1
*T. erythraeum*	0.051/0.061	0.24/0.24	Hydrogen-carbonate: L-glutamineamido-ligase	R00575/EC 6.3.5.5
			Dimethylallyl-diphosphate: isopentenyl-diphosphate dimethylallyl transtransferase	R01658/EC 2.5.1.1
*A. variabilis*	0.051/0.061	0/0.96	4-Methyl-2-oxopentanoate: NAD+ oxidoreductase	R01651/EC 1.2.1.25
			1-Deoxy-D-xylulose-5-phosphate pyruvate-lyase	R05636/EC 2.2.1.7
Anabaena	0.051/0.061	0/0.96	3-(4-Methylpent-3-en-1-yl)-pent-2-enedioyl-CoA hydrolyase	R03493/EC 4.2.1.57
			1-Deoxy-D-xylulose-5-phosphate pyruvate-lyase	R05636/EC 2.2.1.7

For each species the table shows the predicted reactions whose knockout is expected to provide maximal astaxanthin production rate, the expected astaxanthin production rate, and the expected decline in growth rate.

Comparing the predictive performance of MIRAGE on reconstructing the metabolic network of *E. coli *with that of Model SEED [22] has shown a marked advantage to the former. While the number of core reactions considered by MIRAGE and the SEED algorithm in the reconstruction of a metabolic network model of *E. coli *is close (812 and 826 reactions for MIRAGE and SEED, respectively), the number of predicted gap-filling reactions by MIRAGE was 62, in comparison to only 10 by SEED. This results from MIRAGE's aim to resolve all gap-filling problems instead of just enabling biomass production as performed by SEED. The precision of MIRAGE's predictions was significantly higher than that of SEED, reaching 41.9% for MIRAGE versus 10% for SEED. Re-running MIRAGE given the very same definition of a biomass reaction used in the SEED reconstruction of *E. coli*'s model (rather than the biomass definition taken from iAF1260) still resulted in a higher number of 76 predicted gap-filling reactions, with a significantly higher precision of 34.2% than that achieved by SEED.

### Applying MIRAGE to reconstruct metabolic network models for cyanobacteria

To demonstrate the utility of MIRAGE, we applied it to reconstruct genome-scale metabolic network models for 36 cyanobacteria for which genomic data are available to define core reactions sets. Our analysis spans all cyanobacteria for which enzyme annotations are available in KEGG, including *Synechocystis, Synechococcus, Cyanobacteria, Prochlorococcus, Anabaena*, and so on [49]. For all species, we considered the same biomass function, obtained from a previously reconstructed model of *Synechocystis *sp. PCC 6803 [50], assuming that CO_2 _is the sole carbon source. Due to lack of comprehensive gene expression for most cyanobacteria species, we utilized here only phylogenetic data (considering all species in KEGG) to define reaction weights.

**Figure 3:**
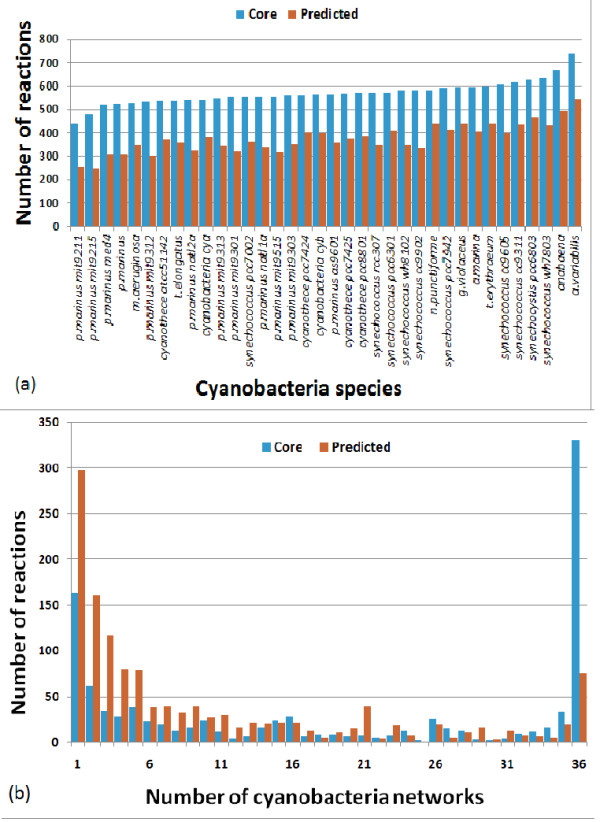
**Statistics on MIRAGE's reconstructed cyanobacteria metabolic networks**. **(a) **The number of core reactions (blue) and predicted gap-filling reactions (red) in the various reconstructed cyanobacteria models. **(b) **A histogram of core reactions (blue) and predicted gap-filling reactions (red) that participate in different numbers of reconstructed cyanobacteria models. As shown, cyanobacteria network cores consist of many reactions that are known to exist in all 36 species, while many of the predicted gap-filling reactions are species-specific.

The average size of a core reactions set for a cyanobacteria network is 570 reactions (Figure 3a), out of which, 331 reactions belong to all of the 36 network cores (Figure 3b). The high degree of similarity between the reaction cores of the various cyanobacteria species reflects the current knowledge on common metabolic processes across these species, obtained mostly from sequence comparisons. These shared core reactions belong to highly conserved metabolic pathways, such as glycolysis, gluconeogenesis, and the TCA cycle among others. MIRAGE's predictions extend these networks in a species-specific manner, with many reactions predicted to belong to a small number of species (Figure 3b). These species-specific reactions belong to more peripheral pathways, for example, diterpenoid biosynthesis, fluorene degradation and others.

**Figure 4:**
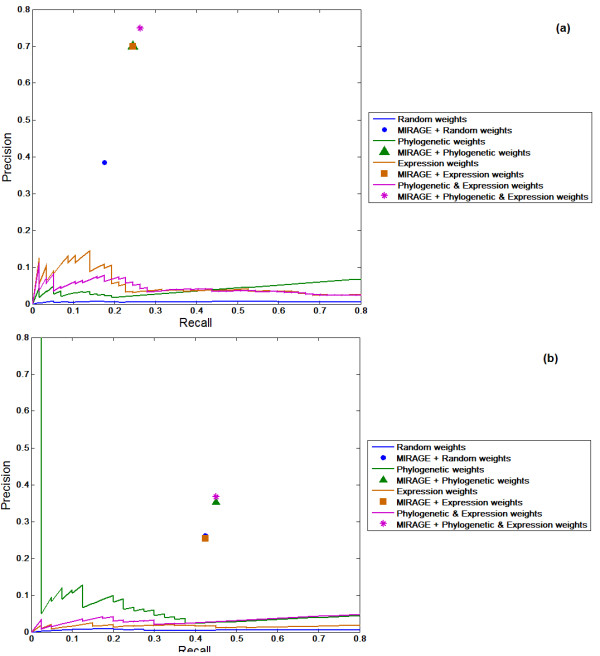
**MIRAGE's predictive performance on reconstructing a known metabolic network of the cyanobacteria *Synechocystis *sp**. PCC6803. **(a) **Metabolic network after Knoop *et al. *[50]; **(b) **metabolic network after Montagud *et al. *[51,52]. The precision and recall of MIRAGE is marked with a star symbol. The precision and recall of a variant of MIRAGE that does not utilize functional-genomics data is marked with a circle. The predictive performance of the functional genomic data (without metabolic flux analysis) is shown by the purple line. The performance of random predictions of gap-filling reactions is colored blue.

To evaluate the performance of MIRAGE in reconstructing cyanobacteria models, we compared a reconstructed network model for *Synechocystis *sp. PCC 6803 with the manually curated models of Knoop *et al. *[50] and iSyn811 [51,52]. In this case, MIRAGE was applied to reconstruct a *Synechocystis *model by further utilizing gene expression data obtained from Tu *et al. *[53] as part of the reconstruction process (Materials and methods). The comparison shows a predictive precision of 70% and recall of 24.6% for the Knoop *et al. *model [50] and precision of 37.5% and recall of 45% for iSyn811 [51,52]. These results are significantly better than random sampling (hyper-geometric *P*-values are 2.99 × 10^-27 ^and 3.59 × 10^-31 ^for Knoop *et al*.'s model and iSyn811, respectively). Again, we find that the predictive performance of either the functional genomics data or the flux analysis alone is far worse (Figure 4). A comparison with the predictive performance of Model SEED was not possible in this case, as the SEED algorithm was not applied to reconstruct cyanobacteria models (focusing only on well-studied and annotated genomes). As a further evaluation criterion, we performed a BLAST [54] search of the known enzyme sequences catalyzing the predicted gap-filling reactions in other species against the genomes of the corresponding cyanobacteria. Reassuringly, we found that the resulting BLAST *E-*scores show significantly higher sequence similarity for the set of predicted reactions in comparison to a random set of reactions (*t-*test of 1.04 × 10^-74^). Moreover, 20.3% of predicted reactions showed E-values below 10^-100^, compared to 9.7% of randomly sampled reactions, testifying the overall correctness of the predicted set of reactions.

As a further evaluation of our reconstructed *Synechocystis *model, we applied it to predict gene knockout lethality data provided by [50]. We find that the prediction performance of our model is comparable with that of Knoop *et al. *(Supp. Table 3 in Additional file 1): out of 39 genes known to be non-essential, Knoop *et al. *correctly predicted 35, while our model correctly predicts 38. Out of 11 known essential genes, Knoop *et al. *correctly predicted 7, while our model correctly predicts 6. The fact that our automatically generated model reaches a similar level of prediction performance to that of a manually curated model demonstrates the applicability and importance of our model reconstruction approach.

### Utilizing the reconstructed cyanobacteria networks for metabolic engineering

To demonstrate the applicability of the reconstructed cyanobacteria networks, we applied a computational metabolic engineering approach called Optknock [55] on these networks to rationally design genetic modifications that would increase the production of astaxanthin, which is a powerful antioxidant belonging to the carotenoid family. These metabolites are known to be produced by various cyanobacteria [56,57]. Optknock works by searching for gene knockouts that would couple the maximal production and secretion of a molecule of interest with a naturally selected trait of maximizing growth rate. Notably, 24 of the original core networks extracted from KEGG include an astaxanthin production reaction, though only 8 of these are not dead-end. In contrast, 25 of the network models reconstructed by MIRAGE have a functional astaxanthin production pathway, amenable for Optknock analysis.

The application of Optknock for astaxanthin production identified double gene knockouts in 15 species that are expected to lead to astaxanthin secretion (Table 1). For 12 out of these, Optknock predicts the knockout of dimethylallyl-diphosphate: isopentenyl-diphosphate dimethylallyl transtransferase (EC: 2.5.1.1), which consumes an essential precursor for astaxanthin biosynthesis (1-hydroxy-2-methyl-2-butenyl4-diphosphate). The maximal achievable astaxanthin production rate reaches 2.18 μmol gDW^-1 ^h^-1 ^in Prochlorococcus marinus 9601, representing a carbon utilization of 40% for astaxanthin production (considering a CO_2 _uptake rate of 0.22 mmol gDW^-1 ^h^-1 ^[50]). This utilization of CO_2 _to produce astaxanthin is predicted to reduce growth rate by 57% relative to the wild-type Prochlorococcus strain (Table 1).

## Conclusions

Our paper presents a novel method, MIRAGE, for reconstructing metabolic network models by integrating metabolic flux analysis and functional genomics data to resolve network gaps. MIRAGE was validated based on a comparison of its predictions with manually curated metabolic networks for *E. coli *[46] and *Synechocystis *sp. PCC 6803 [50-52]. Then it was applied to reconstruct metabolic network models for an ensemble of cyanobacteria, with the resulting networks shown to be amenable for metabolic engineering applications of astaxanthin secretion.

Our results show that functional genomics data enable the marked improvement of gap-filling in metabolic networks. Furthermore, we show that the integration of more than one type of functional genomics data can further improve the performance of MIRAGE. Naturally, MIRAGE can be extended to account for additional functional genomics data, including protein-protein interactions and genomic context data, which were previously used for the identification of missing gene annotations in metabolic networks [38]. Metabolomics data can also be integrated within MIRAGE, to enable the definition of a metabolite core, consisting of metabolites that are known to be synthesized, and hence corresponding pathways that connect them to the rest of the network must be identified [26].

Several existing gap-filling methods work by searching for a minimal set of missing reactions that would enable the network to perform a certain task [22]. MIRAGE extends upon these methods by enabling the identification of pathways that are not necessarily minimal in size, if supported by functional genomics data. However, MIRAGE is still limited in being unable to predict the presence of alternative pathways, in case either one is sufficient to fulfill its defined objectives. This may explain the relatively low recall levels achieved by MIRAGE and the other tested approaches. For example, this was demonstrated in Figure 1, where reaction R6 will not be predicted for gap-filling, as an alternative pathway that fulfills the required metabolic objectives was chosen. The identification of alternative pathways based on more complex integration of functional genomics data with metabolic flux analysis is currently an open challenge for all known gap-filling algorithms. An additional limitation of MIRAGE is that it does not explicitly account for thermodynamic considerations as part of the network reconstruction process. Future implementation of this approach may formulate additional thermodynamic constraints as part of the model consistency check, as suggested in Thermodynamic Metabolic Flux Analysis (TMFA) [58] (which would require further speedups to obtain reasonable running times).

Metabolic models generated by automated methods such as MIRAGE should be regarded as first draft models, requiring further manual curation to bring them up to comparable level with standard manually curated models. The growing interest in reconstructing metabolic network models for hundreds of species raises the challenge of developing improved such gap-filling approaches that could speed up the reconstruction process, while the approach presented here shows a marked improvement in this direction over the state-of-the-art, supporting the advantage of integrating functional genomic data as part of model reconstruction. We expect MIRAGE to be used for automatic reconstructions of many other species, leading to a significant boost in the understanding of their metabolism.

## Materials and methods

### Step I: calculation of functional genomics weights

Binary vectors describing reaction phylogenetic profiles were acquired from KEGG. A phylogenetic weight for each non-core reaction is defined as the maximal Jaccard similarity with a phylogenetic profile of a core reaction that shares a metabolite substrate with the reaction at hand. The Jaccard values are normalized based on the frequency of appearance of the shared metabolite in the universal reaction database (see Additional file 1 for details).

An expression weight for a given non-core reaction is computed by evaluating the correlation between profiles of genes that may potentially code for an enzyme catalyzing the reaction at hand and expression profiles of genes associated with neighboring reactions. Specifically, for each gene in the genome of the target species, we compute the average Pearson correlation between its expression profile and profiles of genes associated with neighboring reactions, with the expression weight defined as the maximal such correlation obtained. All Pearson correlations are normalized by the frequency of appearance of the connecting metabolites (as done above).

The distribution of phylogenetic and expression weights are normalized to having the same mean and standard deviation. Final edge weights are defined based on the sum of normalized phylogenetic and expression weights. Reactions for which either weight is missing are assigned the median normalized value.

### Step II: finding gap-filling reactions supported by functional genomics weights

First, we create a random reaction pruning list by iteratively sampling the next reaction with probability proportional to its weight normalized by the sum of weights of the remaining non-sampled reactions. Next, we scan through the obtained reaction list and try to remove each reaction in turn from the model, as long as the resulting model remains consistent. The consistency check involves verifying that: (i) each core reaction can carry non-zero flux under steady-state and reaction directionality constraints; (ii) there can be non-zero flux through the biomass reaction; and (iii) the growth-dilution of each metabolite in the network is accounted for. Once we finish scanning through the pruning list, we are left with a minimal functional model. We repeat the random pruning procedure 500 times and count the number of times that each non-core reaction appeared in the final model. Finally, we order the non-core reactions based on their frequencies (from low to high) and repeat the pruning step to obtain the final model.

The details of the above and the implementation of the various speed-up techniques that makes this algorithm computationally tractable are described in Additional file 1. The implementation of MIRAGE is available at [44].

## Abbreviations

KEGG, Kyoto Encyclopedia of Genes and Genomes; MBA, Model Building Algorithm; MIRAGE, MetabolIc Reconstruction via functionAl Genomics; SBML, Systems Biology Markup Language.

## Authors' contributions

EV and TS conceived the research and wrote the paper. EV performed the computational analysis. Both authors read and approved the final manuscript.
